# Sphingolipid Metabolism as a New Predictive Target Correlated with Aging and AD: A Transcriptomic Analysis

**DOI:** 10.3390/medicina58040493

**Published:** 2022-03-30

**Authors:** Simone D’Angiolini, Luigi Chiricosta, Emanuela Mazzon

**Affiliations:** IRCCS Centro Neurolesi “Bonino-Pulejo”, Via Provinciale Palermo, Contrada Casazza, 98124 Messina, Italy; simone.dangiolini@irccsme.it (S.D.); luigi.chiricosta@irccsme.it (L.C.)

**Keywords:** Alzheimer’s disease, sphingolipid metabolism, aging, transcriptome, interactome

## Abstract

*Background and objectives*: Alzheimer’s disease (AD) is the most common form of dementia characterized by memory loss and executive dysfunction. To date, no markers can effectively predict the onset of AD and an early diagnosis is increasingly necessary. Age represents an important risk factor for the disease but it is not known whether it is the trigger event. *Materials and Methods*: We downloaded transcriptomic data related to post-mortem brain of thirty samples gathered as young without AD (Young), old without AD (Old), and old suffering from AD (OAD) groups. *Results*: Our results showed that steroid biosynthesis was enriched and associated with aging, while sphingolipid metabolism was related to both aging and AD. Specifically, sphingolipid metabolism is involved in the deregulation of *CERS2*, *UGT8,* and *PLPP2*. These genes are downregulated in Young and Old groups as compared with upregulated between Old and OAD groups. Moreover, the analysis of the interaction networks revealed that GABAergic synapse and Hippo signaling pathways were altered in AD condition along with mitochondrial metabolism and RNA processing. *Conclusions:* Observing the particular trend of genes related to sphingolipid metabolism that are downregulated during normal aging and start to be upregulated with the onset of AD, we suppose that sphingolipids could be early markers for the disease.

## 1. Introduction

Alzheimer’s disease (AD) is the most common form of dementia, and it is estimated to be responsible for 60–70% of total cases around the world [1]. Among developed nations, approximately 1 out of 10 aged persons (age ≥65 years) has some degree of dementia, while more than a third of very elderly people (age ≥85 years) may have symptoms and signs related to dementia [2]. AD is a chronic disease whose effects include loss of memory, language and cognition, changes in behaviour and ultimately death. Changes related to language and vision often precede memory loss and executive dysfunction [3]. Characteristic signs that identify AD include β-amyloid (Aβ) plaques and neurofibrillary tangles (NFTs); however, although extensive research has been conducted in recent decades, the exact role of these proteins is still elusive [4]. Amyloid pathogenesis starts with altered cleavage of an integral protein on the plasma membrane (*APP*). Cleavage by β-secretases (BACE1) and γ-secretases results in insoluble Aβ fibrils that alter the normal synaptic signalling and aggregate into plaques [5]. AD is derived from excessive production and reduced disposal of Aβ [6]. Another key role is played by tau protein. Phosphorylated tau is involved in axonal transport and either polymerization or depolymerisation of microtubules of the neuronal cytoskeleton. In AD, there are high levels of the hyperphosphorylated form of tau protein that aggregates to neurofibrillary tangles, and this can disrupt the physiological functioning of neurons [7]. AD does not usually manifest in young individuals (age at onset <60 years) and often in these cases genetic involvement is considered. In addition to *APP* [8], mutations in *PSEN1* and *PSEN2* that encode, respectively, for presenilin 1 and 2 [9,10] responsible for catalytic subunit of the γ-secretase complex [11], are associated with AD. A genomic analysis can associate mutations to these genes through copy number variation (CNV) inspections [12] or genome-wide association study (GWAS) [13]. Nevertheless, AD is a multifactorial disease and many works have reported that AD is associated with changes in the expression of numerous transcripts [14,15]. A recent study by Peng et al. observed that the gene expression in AD was similar to those of a subpopulation of healthy people (aged 40 to 80) [16]. An interesting manuscript by Xia et al. provided an overview about the connection between aging and AD and confirmed that AD was similar to accelerated aging processes at the molecular level, including DNA methylation, histone modifications, and ncRNA and RNA modifications [17]. Considering that AD as an exacerbation of processes already present during aging, it can be difficult to identify early markers related only to the presence of the disease. Nevertheless, many molecules are under investigation to become possible predictive targets of AD. The involvement of sphingolipids has been extensively studied and, in several works, it has been related to AD [18,19]. Sphingolipids are complex lipids that have a major role in lifespan and regulating development. They are also involved in cell signalling [20,21]. Deregulation in sphingolipid metabolism is directly connected with an increase in the risk and progression of age-related neurodegenerative diseases [22,23]. Jove et al. observed that low sphingolipid levels in plasma seemed to be an important element for healthy aging [24]. In the brain, we have a similar situation because, at low levels, sphingolipids promote cell division and survival, whereas, at higher levels, they inhibit cell division and promote differentiation. Their increase can induce apoptosis and cellular disfunction [22]. Many analyses based on different omics such as lipidomics and metabolomics have highlighted that molecules involved in sphingolipid metabolism, especially ceramides, were altered early in AD. Indeed, sphingolipids contribute to neuropathological alterations associated with AD, including neurodegeneration, tau formation, and Aβ production [25]. In addition, the levels of ceramide also increase in the cerebrospinal fluid of patients with AD [26].

Herein, we chose to study AD under transcriptomic profiles to observe how transcript levels change in normal aging as compared with AD pathology. The different comparisons can help us to find some markers with expression levels specifically related to AD presence and distinguishable from expression levels related to normal aging. For this work, we analysed transcriptomic data of post-mortem brain samples retrieved from the Gene Expression Omnibus [27] (GEO) repository. The transcriptomic data were relative to three groups of people: (1) healthy young people (Young group), (2) healthy old people (Old group), (3) old people suffering from AD (OAD group). Inclusion of the Young group in the work made it possible to compare the normal trend of gene expression in aging with the trend related to the presence of AD.

## 2. Results

**Figure 1:**
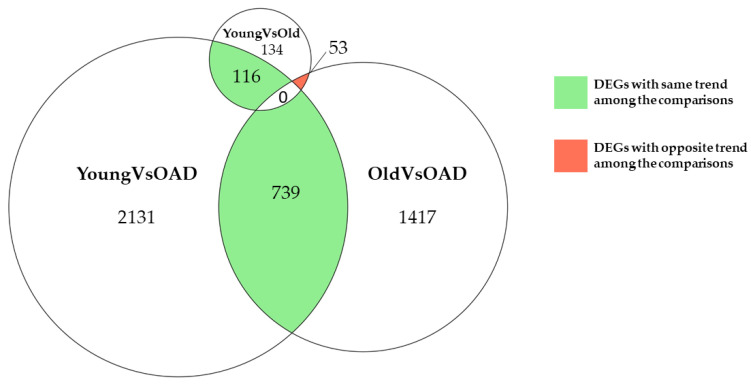
Venn diagram distribution of DEGs between Young, Old, and OAD groups. Green represents all the DEGs that have the same trend among the different comparisons. Conversely, red represents the DEGs that have the opposite trend among the comparisons. Numbers under the name of comparisons are related to the DEGs found exclusively in that comparison.

Young and Old groups were compared to the OAD group to see which and how many genes were differentially expressed. Only protein-coding genes were considered for all the comparisons. The YoungVsOAD comparison resulted in 2986 differentially expressed genes (DEGs), among which 2091 DEGs were downregulated and 895 DEGs were upregulated (Table S1). The OldVsOAD comparison resulted in 2209 DEGs, among which 1304 DEGs were downregulated and 905 DEGs were upregulated (Table S2). Additionally, the Young and Old groups were compared (YoungVsOld) to discriminate the altered processes related to AD from those associated with aging, The YoungVsOld comparison resulted in 303 DEGs, among which 275 DEGs were downregulated and 28 DEGs were upregulated (Table S3). The DEGs obtained from all the comparisons were crossed, as shown in Figure 1, to see which and how many of them were shared among the comparisons; 739 DEGs were found to be common among YoungVsOAD and OldVsOAD and these DEGs were attributable to the presence or absence of AD. Interestingly, all of these common genes had the same trend in both the comparisons; 116 DEGs were common to YoungVsOAD and YoungVsOld comparisons, so it is reasonable to suppose that these DEGs are related to aging. Again, all the common genes had the same trend in both the comparisons. There were 53 DEGs found to be shared by YoungVsOld and OldVsOAD comparisons, but all these DEGs showed an opposite trend among the comparisons. No gene was differentially expressed in all three comparisons simultaneously. This result is important because it shows that there is no DEG that is relevant to the presence or absence of AD and also involved in age difference.

Comparing all transcriptomic profiles, we had a high number of DEGs, therefore, we focused our attention on the genes related to key pathways with important roles for the shared conditions among the comparisons. We chose to analyse the enriched pathways among the DEGs common to different comparisons. The enriched pathways allowed us to identify key pathways that were significantly deregulated rather than the scenario of all the pathways. Among the 116 DEGs common to the YoungVsOAD and YoungVsOld comparisons, the only enriched KEGG pathway was “steroid biosynthesis” (hsa00100) and the involved altered DEGs were *DHCR24*, *FDFT1* and *SQLE*. Among the 53 DEGs common to the YoungVsOld and OldVsOAD comparisons, the only enriched KEGG pathway was “sphingolipid metabolism” (hsa00600) and the involved altered DEGs were *CERS2*, *UGT8*, and *PLPP2*. These results are shown in Table 1. Additionally, in Table 2, we summarize the biological function of each gene explored in the context of our study.

**Figure 2:**
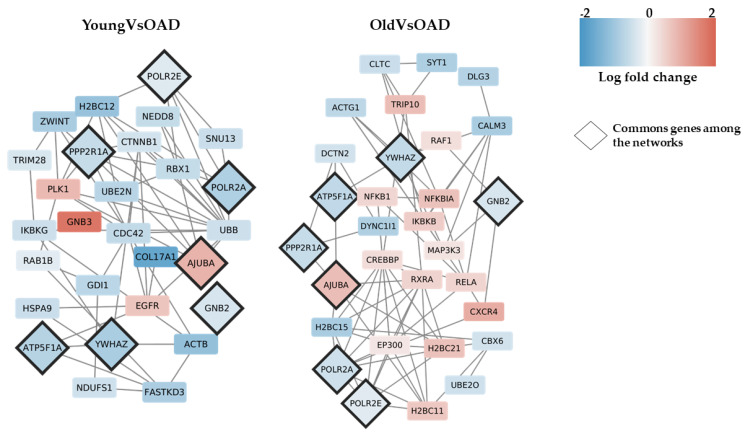
The network analysis of DEGs with the highest betweenness centrality. 29 and 30 are the DEGs with the highest betweenness centrality, respectively, in YoungVsOAD and OldVsOAD. Blue represents the downregulated DEGs and red represents the upregulated DEGs. The seven DEGs common to both networks are represented as rhombus.

The analysis of enriched pathways for the 739 DEGs common to YoungVsOAD and OldVsOAD resulting in 25 enriched pathways. To inspect all the relationships of all the DEGs found in this high number of pathways without losing any biological function, we chose to perform an interactomic analysis. This type of analysis studies the interactions of the proteins coded by out transcripts. Thus, we explored the map of the interactions among all our DEGs to filter the most central ones and detect key genes in AD condition. The dataset of STRING relative to human protein interactions was downloaded to filter out the connections relative to our DEGs and build the networks for comparisons of YoungVsOAD and OldVsOAD. For the network’s construction, only the connections obtained by “experiments” and “database” as source were considered and all the DEGs without connection were excluded. The networks were exported onto Cytoscape to analyse them. The YoungVsOAD comparison resulted in a network with 1387 nodes and 13,388 connections, whereas the OldVsOAD comparison resulted in a network with 727 nodes and 2964 connections. We discriminate the most central nodes by normalizing the values of the betweenness centrality through z-score and keep the nodes outside the 97.5% distribution (z-score > 1.96). The betweenness centrality associated with each node is a score based on the number of shortest paths that pass through that node. At the end of the analysis, 29 DEGs were observed as most central nodes in the YoungVsOAD comparison and 30 DEGs in the OldVsOAD comparison, which are represented in Figure 2.

Interestingly, *AJUBA*, *ATP5F1A*, *GNB2*, *POLR2A*, *POLR2E*, *PPP2R1A*, and *YWHAZ* are common central nodes between the two networks and Table 3 summarizes their statistics.

With the list of DEGs related to enriched pathways or central in the interaction networks it was possible to explore the processes related to aging, AD, or both.

## 3. Discussion

AD is the most common form of dementia and millions of people worldwide suffer from it. We explored which pathological processes were related to aging or AD by studying how they changed among different conditions. By studying different conditions it was possible to find markers that could be related only to the onset of AD pathology and not confused with normal aging expression. For this reason, we studied AD under transcriptomic profiles to compare the expression levels in aging and AD conditions. The study of different comparisons highlights the elements related to one or shared by different conditions. In this way, we collected 30 samples which were split into three different groups: Young, Old, and OAD. The YoungVsOld comparison shows how aging evolves. In addition, since the mean age of the OAD and Old groups were comparable, the OldVsOAD comparison could highlight changes due to the onset of AD. The YoungVsOAD comparison was intriguing. Indeed, here, we see the combined effects of aging and AD. For this reason, the YoungVsOAD comparison did not show changes in Young and OAD groups that were negatively correlated [35]. Additionally, our samples were almost exclusively males, and therefore the three different cohort were also homogenous with respect to gender. Indeed, males and females show many differences both in the development and progression of AD [36]. Thus, the homogeneity of gender in the cohorts is necessary to compare them. Analysing the list of shared DEGs among the YoungVsOld, OldVsOAD and YoungVsOAD comparisons, we observed which were the most altered pathways. Studying the 116 DEGs common between the YoungVsOAD and YoungVsOld comparisons, represented in Figure 1, we can observe that all of these have the same trend, so they are up- or downregulated in both comparisons. Curiously, Figure 1 highlights that these DEGs are not downregulated in OldVsOAD. Thus, it is reasonable to attribute their expression just to aging. To establish a common role among the 116 DEGs, we inspected the pathways in which they were involved. As shown in Table 1, steroid biosynthesis is the only pathway enriched in KEGG. Schumacher et al. showed a decrease in the level of neurosteroids during aging exposed to dementia forms such as AD, where we found lower levels of these molecules. This was in accordance with our results where we observed decreased expression levels of the genes *DHCR24*, *FDFT1* and *SQLE*, as shown in Table 1, that are involved in the steroid biosynthesis pathway. *DHCR24* can regulate the metabolism of desmosterol to cholesterol and has been shown to impede the formation of amyloid-β [28,30]. *SQLE* squalene is closely related to cholesterol synthesis which is known to influence important AD processes such as Aβ production and accumulation [30]. *FDFT1* is responsible for the synthesis of squalene catalysing this biosynthesis [29], and therefore it plays a key role upstream in this pathway. Analysis of these DEGs and the comparisons from which they have been found leads us to conclude that decreased levels of neurosteorids play an important role as a risk factor for AD considering that low levels are associated with an increase in Aβ and hyperphosphorylated tau.

There were 53 DEGs shared between the YoungVsOld and OldVsOAD comparisons, as shown in the Figure 1, all have opposite trends among the comparisons. The only enriched pathway among these 53 DEGs is relative to sphingolipid metabolism, as Table 1 describes. The two central bioactive lipids in sphingolipid metabolism are ceramide and sphingosine-1-phosphate (S1P). Ceramide is closely related to AD because its intracellular levels can regulate Aβ generation [37], contributing to stabilising the APP cleaving enzyme 1 (BACE1) [38]. Ceramide is considered to be a core element in the sphingolipid metabolism pathway because all sphingolipids are synthesized from ceramide [39]. A low level of ceramide has trophic effects promoting cell survival and division and is beneficial for development of neuronal cells [40,41]. In embryonic rat brain neurons it has been observed that high concentrations of C2-ceramide induced apoptosis and low concentrations promoted survival of cultured cells [42]. In plasma, high levels of peripheral ceramides have been associated with an increased risk of AD and memory impairment [25].

Among the 53 DEGs mentioned above, the three genes shown as DEGs are *CERS2*, *UGT8*, and *PLPP2*. *CERS2* is ceramide synthase that catalyzes the transfer of the acyl chain from acyl-CoA to a sphingoid base. The statistics related to these genes are shown in Table 1. *CERS2* plays an important role in the synthesis of ceramide, regulating the levels of myelin-specific sphingolipids galactosylceramide and sulfatide that are directly involved with myelin sheath architecture and motor neuron functions [31]. *UGT8* deals with catalysing the transfer of galactose to ceramide [32]. *PLPP2* encodes for a protein of the phospholipid phosphatase family that catalyzes the dephosphorylation of a variety of glycerolipid and sphingolipid including sphingosine 1-phosphate/S1P and ceramide 1-phosphate [33,34]. In our work, we observed that the expression of these three genes involved in ceramide synthesis were downregulated in the YoungVsOld comparison, upregulated in the OldVsOAD comparison, and they were not differentially expressed in the YoungVsOAD comparison. In accordance with our results, the pathway related to sphingolipids, and therefore, to ceramide, during aging undergoes a downregulation. Low levels of ceramides, as previously reported, have been observed in plasma and brain and are related to promotion of cell survival and division. In AD conditions, we have seen how all the DEGs related to sphingolipid metabolism invert their normal trend, starting to be upregulated and this upregulation is related, as previously reported, to cellular dysfunction and apoptosis. Considering that these genes do not result as DEGs in the YoungVsOAD comparison, we can say that, with the onset of AD, the expression of the genes involved in the sphingolipid metabolism pathway returns to be in the same order of magnitude as that of the Young group. Considering that the expression trend of sphingolipid metabolism in AD condition bucks the normal aging trend, we can consider this pathway to be an important early marker for AD pathology. If the Young group had not been included in this transcriptomic analysis, we could not have observed this change in trend that could make this pathway an important marker for AD.

Shared by the YoungVsOld and YoungVsOAD comparisons, as shown in Figure 1, we have 739 common DEGs and among them we want to discriminate the more relevant DEGs that could be interesting in AD pathology. As shown in Figure 2, among the 29 and 30 DEGs were more central, respectively, in the YoungVsOAD and OldVsOAD comparisons, seven DEGs were shared by both networks: *AJUBA*, *ATP5F1A*, *GNB2*, *POLR2A*, *POLR2E*, *PPP2R1A*, and *YWHAZ*. Statistics about these DEGs are reported in Table 3. Considering that these DEGs are central in both networks, we can suppose that all these DEGs are closely involved in AD pathology. *AJUBA* is a protein coding gene that encodes an adapter or scaffold protein which is involved in the assembly of numerous protein complexes and takes part in different cellular processes such as cell fate determination, cytoskeletal organization, repression of gene transcription, mitosis, cell-cell adhesion, as well as cell differentiation, proliferation, and migration [43]. *AJUBA* also plays a key role in the Hippo signalling as an inhibitor [44]. This pathway (hsa04390) was enriched in both the YoungVsOAD and OldVsOAD comparisons, therefore, it represents an important pathway in AD. The core of Hippo signalling is YAP/TAZ that, when it is inactivated through phosphorylation, it goes to cytoplasm, in this case, the Hippo signalling is active. When the Hippo signalling is inactive the non-phosphorylated YAP/TAZ can be translocated to the cell nucleus, and it can bind to TEAD transcription factors and activate the transcription of specific target genes [45]. It has been demonstrated that the Hippo signalling pathway plays an important role in neuroinflammation, neuronal cell differentiation, and neuronal death [46,47]. YAP activation has been implicated in the increase in proinflammatory cytokines, contributing to the progression of AD and was found upregulated in the central nervous system tissue from old AD mice and severe AD patients [48]. Among the seven genes which we are focusing on in this study, *PPP2R1A* and *YWHAZ* are also involved in the Hippo signalling pathway. *YWHAZ* encodes for a protein of the 14-3-3 family, specifically for the isoform zeta/delta (14-3-3ζ). The 14-3-3 proteins are involved in the Hippo signalling pathway because, when YAP is phosphorylated by LATS1/2, it becomes inactive and translocated into the cytoplasm where it is sequestered by these proteins [49]. The *PPP2R1A* gene encodes for PR65, a constant regulatory subunit of protein phosphatase 2 (PP2A). PP2A is also important because it dephosphorylates multiple substrates including tau [50] and it is considering to be the principal dephosphorylation enzyme in the brain [51]. The downregulation negatively affects tau phosphorylation, which acts on AD pathology by promoting an increase in neuroinflammation, since these two processes are closely related [52].

*ATP5F1A* encodes for a subunit of mitochondrial ATP synthase, which is necessary to produce ATP from ADP. The importance of mitochondrial and bioenergetic alterations in AD are well known and are considered to play a key role in the development and progression of the disease [53]. *ATP5A1* is not exclusively involved in the production of ATP and it has also been observed that it is involved in response to neuroinflammation in AD. Downregulation of *ATP5F1A* has been associated with both levels of ATP and neuroinflammation. *GNB2* encodes for guanine nucleotide-binding proteins (G proteins) involved as modulators or transducers in various transmembrane signalling systems. This role is related to many different pathways but the only enriched pathway in the OldVsOAD comparison was the “GABAergic synapse” pathway (hsa04727). Lower levels of GABA have been found in the temporal cortex of AD patients and this indicated a deficient neuronal transmission and synaptic function in AD [54]. *POLR2A* and *POLR2E* are genes that encode for two subunits of RNA polymerase II. *POLR2A* encodes for RPB1, the catalytic subunit of RNA polymerase II necessary for the transcription of mRNAs and many other microRNAs and small nuclear RNAs in the nucleus [55]. *POLR2E* encodes for POL II, the central component of the basal RNA polymerase II transcription machinery and it is also a component of RNA polymerase I and RNA polymerase III [56]. From a recent work about neurons in AD prefrontal cortex based on single-nucleus RNA sequencing, it resulted in a general repression of gene expression [57]. The analysis of our results confirmed that there was observable down expression of these two genes, which are key in the transcriptional process, in the comparisons against OAD; however, downregulation was not observed in the YoungVsOld comparisons, and this suggests that it is not an issue related to normal aging but to the presence of AD.

## 4. Materials and Methods

### 4.1. Data Collection

All data used for our analysis were downloaded from the GEO repository dataset PRJNA413568 [35]. This dataset collects 30 runs containing transcriptomic data related to 3 cohorts: healthy young people (average age 52 years) (Young), healthy old people (average age 68 years) (Old), old people suffering from AD (average age 68 years) (OAD). The cohorts were distributed as follows: 8 Young, 10 Old, and 12 OAD. Among the 30 samples, 27 were males and 3 were females. Each group included just 1 female, respectively. No information regarding other pathologies in progress was included. The RNA was isolated from 20 mg of brain tissue taken from the lateral temporal lobe of each sample and 75-bp single-end sequencing was performed through an Illumina instrument HiSeq 500.

### 4.2. Bioinformatics Analysis

The data from the GEO were all downloaded using fastq-dump version 2.8.0 (https://github.com/ncbi/sra-tools, accessed on 22 February 2021) and the reads quality check was done using FastQC 0.11.9 (Babraham Institute, Cambridge, UK). All adapters and low-quality reads were eliminated with Trimmomatic 0.39 [58]. After the trimming phase, the reads were aligned using the Spliced Transcripts Alignment to a Reference (STAR) RNA-seq aligner 2.7.3a [59]. The genome used as reference for the alignment phase was the GRCh38 human reference genome. Using htseq-count 2.7.3a [60], we obtained the transcription count with which it was possible to carry out the statistical analysis of differentially expressed genes (DEGs) with the DESeq2 library [61] through programming language R (R Core Team). To discriminate the statistically expressed genes, we used the q-value level. Q-value was obtained from the correction of the *p*-value level performed by the false discovery rate method to drop the number of false positives. All the transcripts were filtered out to keep DEGs with q-values lower than 0.05 and related to protein coding genes. This was made by cross-referencing our genes with all the protein coding genes contained in the STRING database [62]. In this work, we identified three lists of DEGs, the first related to the comparison between Young and OAD (YoungVsOAD), the second related to the comparison between Old and OAD (OldVsOAD), and the third related to the comparison between Young and Old (YoungVsOld). The enrichment of pathways and biological processes were performed on DEGs. For the pathways, we used KEGG [63] and for the biological processes we used gene ontology (GO) databases [64,65]. Two networks were built based on the YoungVsOAD and OldVsOAD comparisons to discriminate among many the different genes which were central nodes. The networks of DEGs were obtained using the STRING database. The resulting networks were filtered out to keep just those interactions with “experiment” or “database” as source. The networks were analysed and personalized to better highlight important features using Cytoscape version 3.9.0 [66].

## 5. Conclusions

The analysis of the altered pathways shared by Young, Old, and OAD comparisons shows the steroid biosynthesis pathway is exclusively related to aging, whereas the sphingolipid metabolism pathway is involved both in aging and AD. Additionally, through the interaction networks, we identified GABAergic synapse and Hippo signalling pathways specifically related to AD condition. Noteworthy, mitochondrial metabolism and transcriptomic processing could also be impaired. The originality of this manuscript was the inclusion of a Young group, without which the reverse trend in AD could not be highlighted. Thus, the strength of this analysis is related to the DEGs that have a trend during normal aging and assume an opposite trend with the onset of AD. We found that these DEGs reverse in trend expression and are specific to the sphingolipid metabolism pathway. Indeed, sphingolipid downregulation is related to healthy aging and prevents neuronal death. Conversely, sphingolipid upregulation is related to the onset of AD and leads to apoptosis and neurological impairment.

## Figures and Tables

**Table 1:** Enriched pathways for DEGs commonly expressed among Young, Old, and OAD groups.

Group Comparisons	Enriched Pathway	Involved Gene	Log Fold Change		
			YoungVsOld	OldVsOAD	YoungVsOAD
YoungVsOAD & YoungVsOld	Steroid biosynthesis	*DHCR24*	−0.67	N.S.	−0.85
		*FDFT1*	−0.84	N.S.	−0.60
		*SQLE*	−1.00	N.S.	-1.03
			**YoungVsOld**	**OldVsOAD**	**YoungVsOAD**
YoungVsOld & OldVsOAD	Sphingolipid metabolism	*CERS2*	−1.50	0.68	N.S.
		*UGT8*	−2.10	1.22	N.S.
		*PLPP2*	−1.76	1.23	N.S.

The table shows statistics about log fold changes with the altered pathways shared between YoungVsOld, OldVsOAD, and YoungVdOAD comparisons. The fold changes were computed as log_2_(OAD/Young), log_2_(Old/Young), or log_2_(OAD/Old). All the values are rounded to the second decimal digit. N.S. stands for “not significant” and it is reported if the gene does not differ in a significant statistical manner in that specific comparison.

**Table 2:** Biological function of genes involved in enriched pathways and in common between YoungVsOAD, YoungVsOld, or OldVsOAD.

Gene	Pathway	Biological Function	Reference
*DHCR24*	Steroid biosynthesis	Metabolism of desmosterol and impedes the formation of amyloid-β	[28]
*FDFT1*		Synthesis of squalene	[29]
*SQLE*		Cholesterol synthesis	[30]
*CERS2*	Sphingolipid Metabolism	Synthesis of ceramide	[31]
*UGT8*		Transfer of galactose to ceramide	[32]
*PLPP2*		Catalyzes the dephosphorylation of sphingosine and ceramide	[33,34]

For each gene, the biological function that characterizes its role in aging or AD is reported.

**Table 3:** DEGs with betweenness centrality outside the 97.5% distribution in both networks.

Genes	Log Fold Change		Betweenness Centrality		Degree	
	YoungVsOAD	OldVsOAD	YoungVsOAD	OldVsOAD	YoungVsOAD	OldVsOAD
*AJUBA*	0.91	0.76	0.02	0.04	39	21
*ATP5F1A*	−0.72	−0.61	0.02	0.04	24	19
*GNB2*	−0.36	−0.36	0.02	0.09	51	45
*POLR2A*	−0.80	−0.46	0.03	0.08	126	50
*POLR2E*	−0.28	−0.25	0.02	0.05	140	53
*PPP2R1A*	−0.54	−0.55	0.03	0.05	74	55
*YWHAZ*	−0.84	−0.58	0.04	0.09	27	22

Table shows statistics about seven most central common genes in both networks with log fold changes in both comparisons and betweenness centrality and degree for both networks. The fold changes were computed as log_2_(OAD/Young) or log_2_(OAD/Old). All the values are rounded to the second decimal digit.

## Data Availability

The data presented in this study are openly available in the NCBI Sequence Read Archive at BioProject accession numbers PRJNA413568.

## Supplementary Materials

The following supporting information can be downloaded at: https://www.mdpi.com/article/10.3390/medicina58040493/s1, Table S1: DEGs in YoungVsOAD comparison; Table S2: DEGs in OldVsOAD comparison; Table S3: DEGs in YoungVsOld comparison.
